# Improvement of energy conservation using blockchain-enabled cognitive wireless networks for smart cities

**DOI:** 10.1038/s41598-022-16916-7

**Published:** 2022-07-29

**Authors:** Shalli Rani, Himanshi Babbar, Syed Hassan Ahmed Shah, Aman Singh

**Affiliations:** 1 Chitkara University Institute of Engineering and Technology, Chitkara University, Rajpura, Punjab 140401 India; 2 Chitkara University Institute of Engineering and Technology, Chitkara University, Rajpura, Punjab 140401 India; 3 Department of Computer Science, California State University, Fullerton, CA 92831 USA; 4 Higher Polytechnic School, Universidad Europea del Atlántico, C/Isabel Torres 21, 39011 Santander, Spain; 5 Department of Project Management, Universidad Internacional Iberoamericana, C.P. 24560 Campeche, Mexico; 6 Department of Engineering, Universidad Internacional Iberoamericana, Arecibo, Puerto Rico 00613 U.S.

**Keywords:** Energy infrastructure, Power distribution, Environmental sciences

## Abstract

In Smart Cities’ applications, Multi-node cooperative spectrum sensing (CSS) can boost spectrum sensing efficiency in cognitive wireless networks (CWN), although there is a non-linear interaction among number of nodes and sensing efficiency. Cooperative sensing by nodes with low computational cost is not favorable to improving sensing reliability and diminishes spectrum sensing energy efficiency, which poses obstacles to the regular operation of CWN. To enhance the evaluation and interpretation of nodes and resolves the difficulty of sensor selection in cognitive sensor networks for energy-efficient spectrum sensing. We examined reducing energy usage in smart cities while substantially boosting spectrum detecting accuracy. In optimizing energy effectiveness in spectrum sensing while minimizing complexity, we use the energy detection for spectrum sensing and describe the challenge of sensor selection. This article proposed the algorithm for choosing the sensing nodes while reducing the energy utilization and improving the sensing efficiency. All the information regarding nodes is saved in the fusion center (FC) through which blockchain encrypts the information of nodes ensuring that a node’s trust value conforms to its own without any ambiguity, CWN-FC pick high-performance nodes to engage in CSS. The performance evaluation and computation results shows the comparison between various algorithms with the proposed approach which achieves 10% sensing efficiency in finding the solution for identification and triggering possibilities with the value of $\alpha = 1.5$ and $\gamma = 2.5$ with the varying number of nodes.

## Introduction

In Smart Cities, Wireless networks have progressed quickly in the past years. Considering the natural frequency spectrum’s constraints, it’s clear that the present stagnant frequency allocation techniques won’t be able to accommodate a wide range of new wireless services. Cognitive radio emerges as a solution to this issue of spectral overload by allowing secondary users to utilize the unused portions of licensed spectrum bands^1^. A cognitive radio, as a smart city wireless communication system, is cognizant of the radio frequency environment. It optimizes spectrum utilization by selecting communication characteristics (namely waveform, throughput, and power consumption). Spectrum sensing is among the most important aspects of cognitive radio technology. Attributed to the influence of geographic location on spectrum sensing, only one node’s sensing efficiency cannot be well attained, whereas multi-node CSS can resolve the sensing weaknesses of one node and make preparations by integrating information from various geographic locations^2^. As a result, in cognitive wireless networks, multi-node CSS is a widely utilized sensing technique that can successfully enhance perception reliability, but as the number of sensor nodes grows, so does the energy utilization of the cognitive wireless network, and because the front end of the cognitive network is a rechargeable battery, the energy utilization grows as well. Many enhanced ways are used to increase the lifetime of cognitive wireless networks^3^. The technique of flipping on and off time is being used to prolong the lifetime of the cognitive network and hence reduces the perception efficiency which is ineffective for the spectrum access. Therefore, to lessen the cognitive network workload this technique uses integration between the nodes and the primary users.

In^4^ for achieving the effectiveness, every secondary user doesn’t have to collaborate in the network, and secondary users with the strongest primary user’s signal-to-noise ratio (SNR) engage in spectrum sensing. In reduced SNR cognitive radio (CR) networks, the detecting efficiency for spectrum sensing is examined. However, this publication does not provide analytical formulations for their issues. The process in which the CR nodes choose the appropriate frequency band based on various parameters including the amount of time the spectrum is vacant or the available bandwidth is known as spectrum selection^5^. The process in which CR nodes negotiate communication link with some other CR users is known as spectrum sharing. Blockchain can help to make this spectrum utilization efficient, thus helping directly to support B5G and 6G applications and services. In the previous works, the main problem is that the sensor nodes used for spectrum sensing have restricted energy budgets. They are usually powered by batteries, that must be replenished or refilled (e.g., using solar power) when they run out. Both options are not feasible for some nodes, which means they will be eliminated after their energy supply is exhausted. In^6^ the nodes with low sensing capability would not aid cooperative sensing, but will have an impact on the fusion center’s overall judgment. As a result, nodes with inadequate sensing efficiency should be eliminated from cognitive wireless networks, and only the most consistent nodes should be chosen to engage in cooperative sensing. Nevertheless, the node’s efficiency is not static; it will fluctuate due to internal causes as well as changes in the external environment^7^. As a result, the role of technological changes must be included in the real cognitive wireless radio which thereby enhance the sensing efficient.

### Problem definition

In this article, a security-based CSS using blockchain for smart cities is developed for a cognitive wireless network consisting of a FC and cognitive sensors for spectrum sensing in a defined time period, thereby, we explore the challenge of reducing energy utilization in CSS. The developed technique can adapt to changes in the environment and adjust the number of sensor nodes engaging in CSS in real time, as well as analyze the consistency of sensing nodes in real time and calculate the node’s trust value using an node evaluation algorithm. This technique not only recalls each node’s energy utilization and sensing efficiency, but also its trust value. The trust value is maintained in the blockchain’s consistency list, which is encrypted by the blockchain’s management center to verify that each node conforms to its own trust value without ambiguity. Later, a fusion rule is used by FC to make the ultimate decision on the channel’s occupancy. We employ the energy detector for channel sensing since it is easy to install and does not require existing knowledge of the primary user signal. The energy received on a licensed band is evaluated using this technique. When the level rises beyond a predetermined threshold, it is considered as recognizing primary user transmissions; else, a spectrum hole is verified. The SNR and distance between each node and the FC are assumed to be known.

### Main contributions

The main contributions of the paper are explained as follows:

1. We have developed the secure spectrum sensing method for smart cities based on the performance of minimizing the energy utilization and accuracy of the blockchain-enabled cognitive wireless networks. This method will detect the probability of sensing all the nodes ensuring SNR which is computed by calculating the distance between the FC and each node.
2. The optimum result is based on cooperative sensing which changes the number of nodes participating and the method may adapt to changes in the environment and alter the number of sensor nodes engaged in cooperative sensing in real-time, as well as evaluate the actual dependability of sensing nodes, and the node’s trust value is determined using the node selection algorithm.
3. The optimum conditions acquired are based on the first-order necessary conditions, the algorithm that is developed to find the optimum nodes selection strategy.
4. To gain more energy efficiency, the method not only preserves each node’s energy utilization and sensing efficiency, but also its trust value. The trust value is maintained in the blockchain’s consistency list, which is encrypted by the blockchain’s management center to verify that each node conforms to its trust value without ambiguity.
5. The obtained simulations were utilized to evaluate the suggested algorithm’s performance in terms of finding the solution for the sensing nodes, energy utilization, and average number of sensing nodes.

The paper is represented as follows: section 2 demonstrates the background and motivation of the blockchain and CWN in blockchain; section 3 explains the design analysis of the proposed work; section 4 describes the flowchart for the evaluation and interpretation of nodes; section 5 shows the simulation results and evaluation of the performance; and lastly section 6 concludes the paper.

## Background and motivation

### Blockchain

Digital Ledger Technology (DLT) uses Peer-2-Peer (P2P) communication technologies to preserve records and transactions in a dispersed, decentralized way^8^. Data management and organizing in DLT can be accomplished in a variety of methods. Data can be maintained in a number of ways, including as a linear linked list of blocks or as a Directed Acyclic Graph (DAG)^9^. The term “blockchain” refers to the management of data is in the form of a linear linked list of blocks. One of the most distinguishing characteristics of blockchain is that it fully excludes the need of a trusted third party in the network’s maintenance^10^.

**Figure 1:**
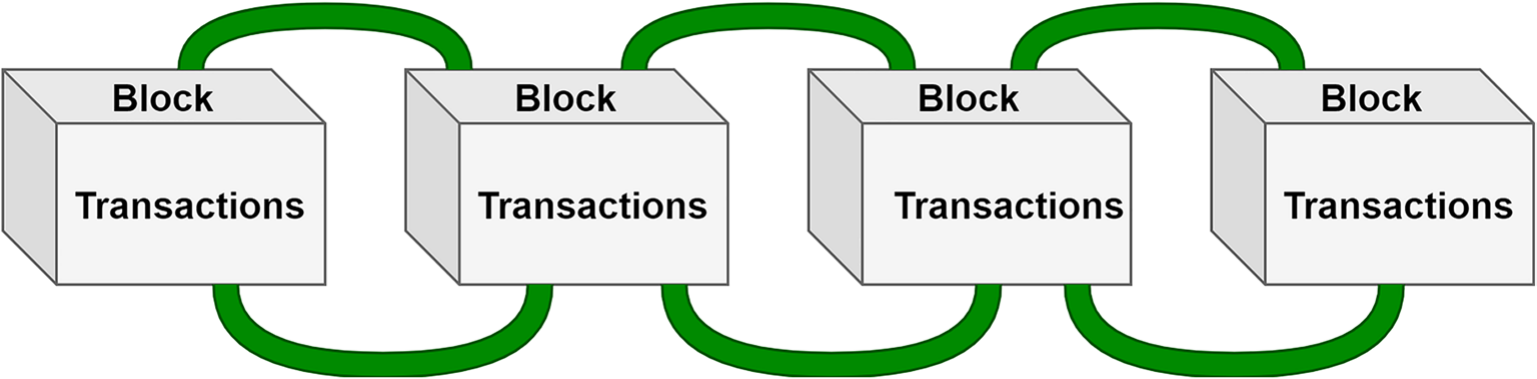
Multiple blocks connected together to form a blockchain network.

A “block” is a building block of a blockchain network where transactions are constructed. Figure 1 depicts a blockchain network with multiple blocks interconnected (each block contains numerous transactions). To prevent tampering, these transactions are put together in a block utilizing cryptographic methods^11^. The blocks are then combined together to form a blockchain^12^. This linkage can be done in a variety of ways. One simple method is to connect these blocks in a straight line. However, there could be concerns with sustainability, quick access to these blocks, and privacy.

**Blockchain is also used from the context of spectrum licensing:**

- **Licensed Spectrum Band:** Only licensed users (also known as primary users) have accessibility to licensed frequency bands, and users without a valid license are not permitted to utilize licensed frequency bands. Users of licensed spectrum willing to obtain it in order to produce more money or to receive a financial incentive from the regulator in the form of lower license costs. When acquiring and exchanging licensed spectrum, cognitive radio (CR) users that want to use these licensed bands take into account a number of factors.
- **Unlicensed Spectrum Band: **Users can use the spectrum without paying any fees or obtaining permission in the unlicensed spectrum concept, enabling anybody to easily utilize any piece of the unlicensed spectrum. Users in the unlicensed spectrum band seek to work together to ensure some quality of service, limit harmful interference, and minimize channel contention. From a licensing standpoint, blockchain can provide a critical part in these spectrum-sharing concepts.

### Cognitive wireless networks in blockchain for smart cities

Constraining spectrum availability only to licensed users is a very ineffective use of resources, as actual dimensions show that such resources are left unused for extended periods^13^. This observation, combined with the advancement of sophisticated nodes aware of exploring licensed spectrum and adapting transmission parameters correspondingly, inspired the concept of cognitive radio, in which the spectrum is made accessible to both licensed (also known as primary)users and unlicensed (secondary/cognitive) users who proactively obtain the sensed spectrum.

**Figure 2:**
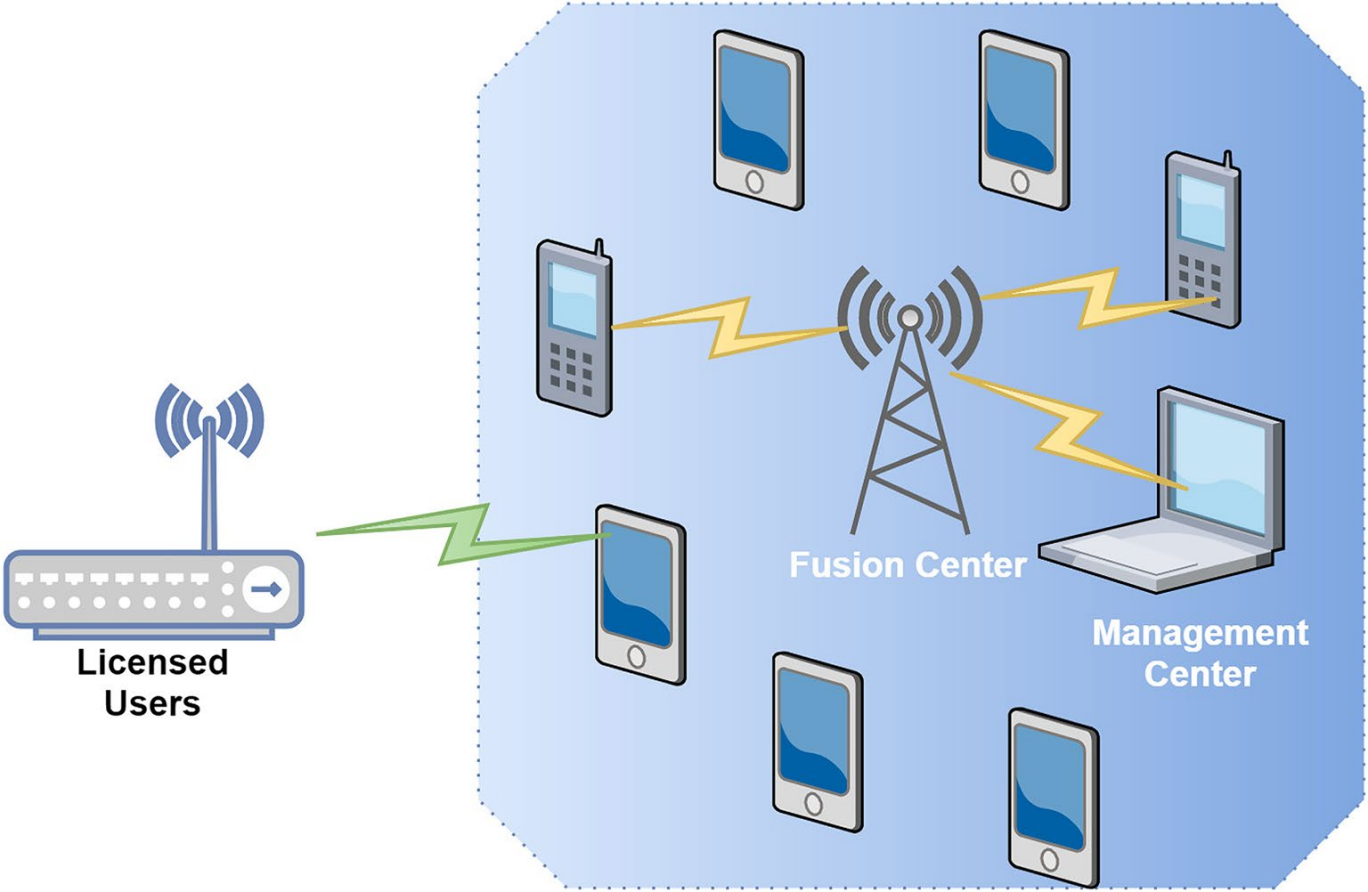
Blockchain-enabled cognitive wireless network.

A cluster of neighboring nodes is formed in blockchain-enabled cognitive radio networks (CRN). These node groups are in charge of allocating radio resources within the group. Well within CRN, the nodes inside each group exchange information they expect to use^14^. This set of resources will be vetted among group members first, but once resources have been allocated without dispute, they will be disseminated and added to the blockchain. The CRN will be able to allocate resources efficiently in this manner. All nodes in the network will be notified of which resources are assigned to which nodes, lowering the likelihood of a CRN clash. Secondly, because blockchain offers a replica of the ledger to every node (in this case, the copy of the ledger will constitute available resources to devices) as well as every CR node would have a broad perspective of assigned network resources, the entire network nodes would be informed of assigned resources amongst these CR nodes^15^.

In this article, we have designed the architecture of blockchain-enabled CWN for Smart cities. The blockchain’s management center, licensed users, FC and cognitive users (various clusters or nodes) are all included in the technique. Some clusters or nodes are in the spectrum detecting mode, while others are in the sleep mode. A centralized CSS mechanism is used in this technique^16^. Only the FC and each node can share resources. Each node sensing data is delivered to the FC for evaluation, and the FC sends information back to each node. Since there is no immediate contact among nodes, a centrally controlled design aids in the efficient processing of data. The FC is accountable for not only communicating with the nodes but also with the blockchain management center, delivering node information to the management center for storing and having the blockchain management center notify the FC with node information.

The blockchain management center and the FC play a significant role in determining which nodes are reliable. Figure 2 shows that not all nodes engage in cooperative sensing at the same time while some of the nodes are nodes tampering^17^. Since the nodes are clustered and the node with the greatest trust value is chosen to engage in cooperative sensing, nodes’ tampering is excluded. The goal is to increase energy efficiency and extend the lifetime of the cognitive network. The energy utilization is lowered based on fulfilling the sensing efficiency.

## Design analysis

**Figure 3:**
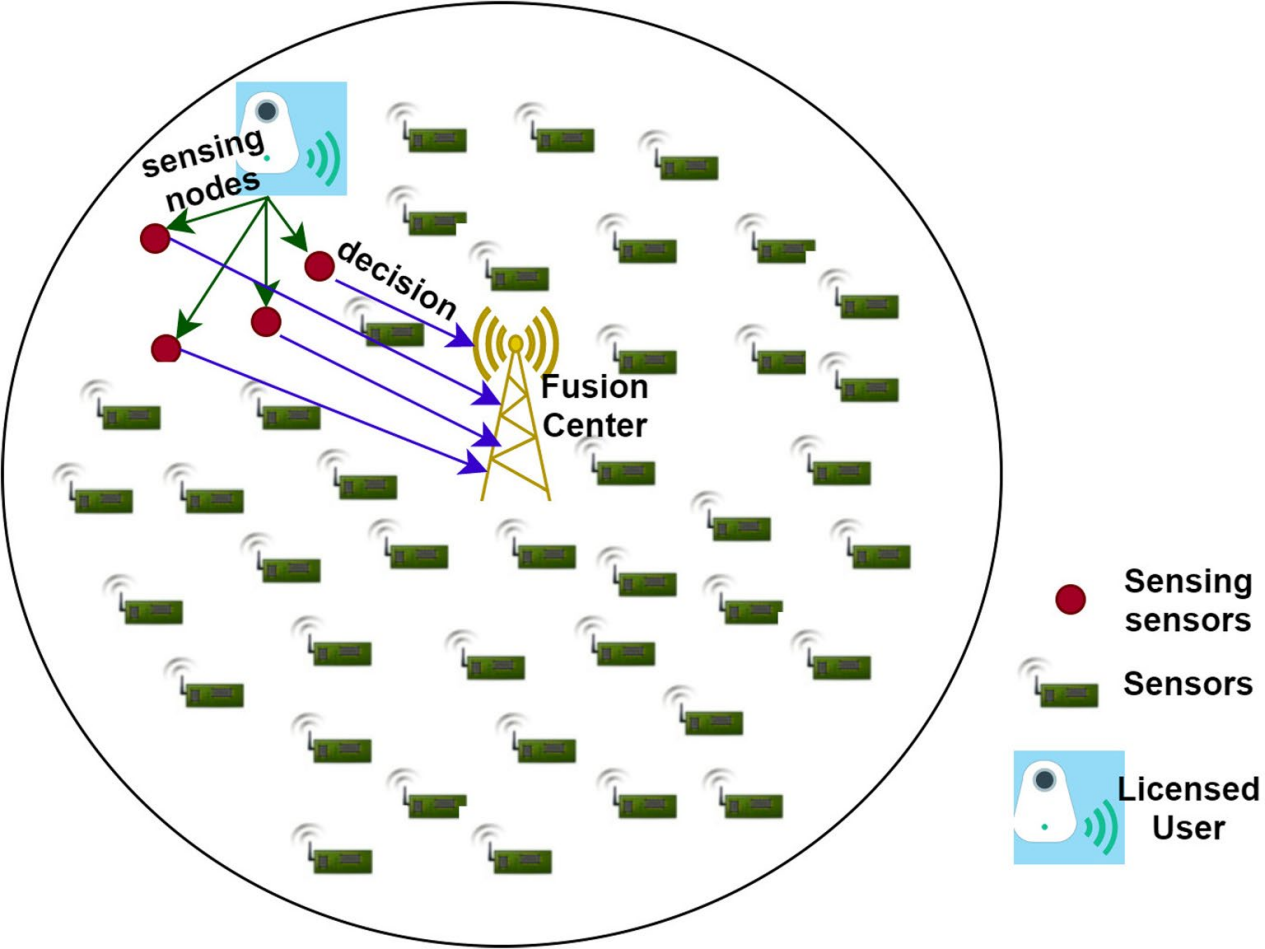
Cooperative spectrum sensing for smart cities.

In Fig. 3, we examine a network with *C* cognitive sensors and an FC. The frames time is denoted by *F*. We consider that almost all cognitive sensors have a similar spectrum sensing time, thus we employ $\varphi$, $\varphi<0<F$ to represent the CSS sensing time. For every user u, $\forall$ u $\in$ C, we presume that *fn* is the Nyquist frequency of the acquired signal from the licensed user^18^. $\mu$ is considered as the manifold of $\frac{1}{fn}$, therefore the total samples are taken as $\varphi \frac{1}{fn}$. Based on the observed scenario, as decided on the status every sensed node has $A_u[m]$ where $m=1,2,3,\dots , \varphi fn$.

In this, every observed scenario comprises two hypotheses. $H_1$, hypotheses signifies that licensed users are in the spectrum detecting mode and $H_0$ hypotheses signify licensed users are in the sleep mode.

1 $$\begin{aligned} H_1 \rightarrow A_u[m]= & {} n_u[m] + p_u[m] \end{aligned}$$

2 $$\begin{aligned} H_0 \rightarrow A_u[m]= & {} p_u[m] \end{aligned}$$

The signal of licensed users at the *u*th sensor is denoted as $n_u [m]$ and is considered to be the process done randomly with the 0th mean and variance $\sigma ^2_{nu}$. Therefore, the noise $p_u[m]$ is a gaussian, independent and similar dispersed random process with the 0th mean and the variance $\sigma ^2_u$. It is presumed that the independent processes are $n_u[m]$ and $p_u[m]$. Using hypothesis $H_1$, we represent u as the licensed user’s acquired SNR determined at the *u*th sensor. The possibility of identifying *Id* and possibility of triggering *Tr* are the possibilities of recognizing a licensed user under $H_1$ and $H_0$, respectively, in node sensing. A cognitive sensor can thus detect whether a node is active or sleep based on these possibilities^19^. An increased Id safeguards a licensed user broadcast from interruption with an unlicensed user broadcast, whereas a reduced Tr allows unlicensed users to access sleep nodes. As a result, a network with an increased Id and a reduced Tr is desired. The following is the decision rule used by the energy detection for the sensor u:

If $$H_0$$, then $$R_u = 0$$;

3 $$\begin{aligned} \in D_u = \frac{1}{\varphi fn} \sum ^{\varphi fn}_{m=1} A_{um}^2 < H_0 \end{aligned}$$

If $$H_1$$, then $$R_u = 1$$;

4 $$\begin{aligned} \in D_u = \frac{1}{\varphi fn} \sum ^{\varphi fn}_{m=1} A_{um}^2 > H_1 \end{aligned}$$

where $\in$ signifies all the cognitive sensors for detection of threshold and $R_u$ signifies the decision made by the node u. As a result, each user transmits a single bit to notify the FC of whether the licensed user signal is 1 or 0. The test constant $D_u$ is a random variable with a chi-square distribution with $2\varphi fn$ degrees of freedom during $H_0$, and a non-central chi-square probability with $2\varphi fn$ degrees of freedom and a non-centrality parameter $2_{yu}$ under $H_1$.

We are assuming the OR rule to be applied for the final decision. In other words, if either of the sensors detects an active licensed user, the final decision proclaims the channel to be congested. We suppose that every CR in the identical channel makes its own decisions. The final decision for *Id* and *Tr* are shown in Eqs. (5) and (6) as below:

5 $$\begin{aligned} Tr (\varphi ) = 1 - \Pi ^C_{u=1} (1- Tr_u (\varphi )) \end{aligned}$$

6 $$\begin{aligned} Id (\varphi ) = 1 - \Pi ^C_{u=1} (1- Td_u (\varphi )) \end{aligned}$$

It is demonstrated that spectrum sensing does not necessitate the participation of all the nodes^2^. For spectrum sensing, our method selects nodes with greater detection probability and shorter distances from the FC. As a result, we can change (5) and (6) with the Eqs. (7) and (8) below. In this $\beta u \in {0,1}$ which indicates the assignment index, 1 and 0 are used for sensing and not sensing the spectrum by the CSS respectively:

7 $$\begin{aligned} Tr (\varphi ) = 1 - \Pi ^C_{u=1} (1- \beta u Tr_u (\varphi )) \end{aligned}$$

8 $$\begin{aligned} Id (\varphi ) = 1 - \Pi ^C_{u=1} (1- \beta u Td_u (\varphi )) \end{aligned}$$

## Proposed Method for Evaluation and Interpretation of nodes

It is crucial to use the node trust value as an essential measure to cooperate in CSS to enhance the safety of CWN. As a result, integrating the node trust value with the basic system design can improve sensing accuracy while reducing energy utilization. To avoid data ambiguity, the blockchain management center can be more efficient^20^. The proposed flowchart enhances the accuracy of sensing and performance of the CWN so, this method begins by estimating the system’s consistency. This estimate is based on accessible statistical information. When an accused node is identified, it generates an instantaneous decision to isolate the node’s sensing data. The method achieves the system’s resiliency but boosts energy utilization, and the effects of global variations on the node are not taken into account. The licensed user’s living conditions have an impact on node sensing. For example, when the licensed user’s location changes, nodes with strong sensing may turn mischievous in the next instant, whereas nodes with low performance become a trusted nodes. As a result, to detect modifications in node status, a real-time evaluation system for nodes must be established. When a node’s efficiency worsens, it can cease detecting work in real-time, and when it improves, it can be moved to work in real-time.

This article establishes an interpretation of nodes and an evaluation of the nodes method to determine and identify nodes more effectively. Before executing spectrum sensing procedures, the CWN determines the consistency of each node, which is based on scientific data. The original aim will continue working whenever the global environment is stable, but when the global environment changes, the node’s consistency must be re-evaluated. To prevent issues, the node’s trustworthiness level is computed using Eq. (9), and the FC creates a nodes list and transmits node data to the blockchain’s management center. The management center effectively delivers node data and is in charge of scheduling nodes to engage in cooperative sensing based on the fusion center’s needs.

9 $$\begin{aligned} y_u = \frac{\sum ^m_{a=1} |L_{u,a}| * l_{u,a}}{\sum ^m_{a=1} |L_{u,a}|} \end{aligned}$$

$y_u$ represents the starting trust value for the $u_{th}$ node, $|L_{u,a}|$ signifies the CSS in the *a*th cycle of sensing of the *u*th node, $l_{u,a}$ denotes the worth value acquired in the *a*th cycle of sensing of the *u*th node. When the $l_{u,a} = 1$; signifies the *u*th node in the *a*th cycle of sensing is reliable with the FC, and $l_{u,a} = 0$; signifies the *u*th node in the *a*th cycle of sensing is not reliable with the FC. The evaluation and interpretation of nodes achieved by Eq. (9) are used to store the value in the blockchain management center. The steps to evaluate and interpretation of nodes is explained as:

- Firstly, check whether the global environment has been modified, if yes, then re-evaluate the trust value of the nodes, otherwise, sensing nodes need not be modified.
- Then, FC will establish the list of nodes’ trust values.
- Later, the blockchain management center is accountable for managing and scheduling nodes.
- Further, adjust the number of sensing nodes and then call the nodes whose trust value is greater than the threshold value to engage in CSS.

Efficiency return value *er*, energy utilization return value *eu*, overall return value *or*, co-efficient of efficiency correction $\rho$, coefficient of energy utilization correction *ec*, and overall correction coefficient *oc* are the three return values and three correction coefficients set. These are computed in the given Eq. (10) for the Efficiency return value *er*:

10 $$\begin{aligned} er = \frac{1}{m} \sum ^m_{a=1} [(1-\beta _a)(\alpha _a * W_C + (1-\alpha _F)P_C)] + \beta _a(\gamma _a * W_C + (1-\gamma _a) * P_C) \end{aligned}$$

In the above Eq. (10), the value of $\beta a$ is either 1 or 0 which indicates that if its 1 means, the licensed user is in the sleep mode and 0 indicates the licensed user is in the active mode, u represents the same as given above, $W_C$ signifies the worth coefficient and $P_C$ signifies the unlawful coefficient. In this equation, $\alpha _a$ and $\gamma _a$ are the weighted coefficients which are represented in Eq. (11).

In $$H_0$$ $$\rightarrow$$ $$\alpha _a$$ $$=$$ 1, $$\beta _a$$ $$=$$ 0, $$H_1$$ $$\rightarrow$$ $$\alpha _a$$ $$=$$ 0, $$\beta _a$$ $$=$$ 0

11 $$\begin{aligned} In H_0 \rightarrow \gamma _a = 0, \beta _a = 1 \quad and \quad In H_1 \rightarrow \gamma _a = 1, \beta _a = 1 \end{aligned}$$

The representation for computation of energy utilization eu is shown in Eq. (12)

12 $$\begin{aligned} eu = \frac{1}{m} \sum ^m_{a=1} [E_WZ_a + E_P(1-Z_a)] \end{aligned}$$

where $E_W$ represents the worth energy utilization which says that the node used for energy utilization is lower than the threshold value; $E_P$ represents the punishable energy utilization which says that the node used for energy utilization is higher than the threshold value. $Z_a$ is the energy return value for the weighted coefficient and its value is denoted in Eq. (13) as: $Z_a = 1$, $\tau _0$, $\sum ^{I_v}_{a,u}$ = 0

13 $$\begin{aligned} Z_a = 0, \tau _0 - \sum ^{I_v}_{a,u} < 0 \end{aligned}$$

where $\tau _0$ represents the threshold of energy utilization in a sensing duration. The overall return value for energy utilization is computed in Eq. (14) as:

14 $$\begin{aligned} or = 0.3er + 0.7eu \end{aligned}$$

This equation describes that 30% of the weight is assigned for the energy utilization return value and the rest 70% of the weight is assigned for the efficiency return value. Thereby, authors have focused on the sensing efficiency while taking into consideration the minimizing of energy utilization. The equation for calculating coefficient of coefficient correction $\rho$ is:

15 $$\begin{aligned} \rho _u = \sum _a (\mu _{a,u} - \beta _a) \end{aligned}$$

The total number of repetitions the *u*th node communicates incorrect information to the FC is represented by the correction coefficient $\rho _u$; $\mu _a$,u shows that in the *a*th sensing cycle, the outcome provided by the *u*th node to the fusion center; $\beta _a$ reflects the outcome of the *a*th sensing cycle’s decision. The energy utilization correction coefficient is denoted by ec is computed in the Eq. (16).

16 $$\begin{aligned} ec_u = \sum _a S_{a,u} * J_{a,u} \end{aligned}$$

The total number of repetitions the *u*th node increases the value of threshold is represented by the energy utilization; the Eq. (17) shows the significance of $J_{a,u}$. In case $J_{a,u} = 1$ means $v_{a,u} - \tau _0 >= 0$ and $J_{a,u} = 0$ means $v_{a,u} - \tau _0 < 0$. $\tau _0$ means the threshold value has been raised for energy utilization and its value is computed where I is the total number of nodes present in the CWN; and $v_{a,u}$ tells that the energy utilized for the *u*th node in the *a*th sensing cycle is shown in Eq. (17).

17 $$\begin{aligned} \tau _0 = \frac{\sum ^I_{u=1} v_{a,u}}{I} \end{aligned}$$

The predefined value for the overall correction coefficient oc is shown in Eq. (18).

18 $$\begin{aligned} oc_u = 0.3\rho _u + 0.7ec_u \end{aligned}$$

$oc_u$ is the overall correction coefficient for the *u*th node which has been acquired by the total weighted count of the efficient and energy utilization correction coefficient. The efficiency has been evaluated by 30% and 70% for the efficient and energy utilization correction coefficient respectively. The trust value of the nodes are computed as shown in Eq. (19).

19 $$\begin{aligned} y_u^{r+1} = y_u^r + (\omega oc - (1-\varphi ) oc_u^r) y_u^r \end{aligned}$$

**Figure 4:**
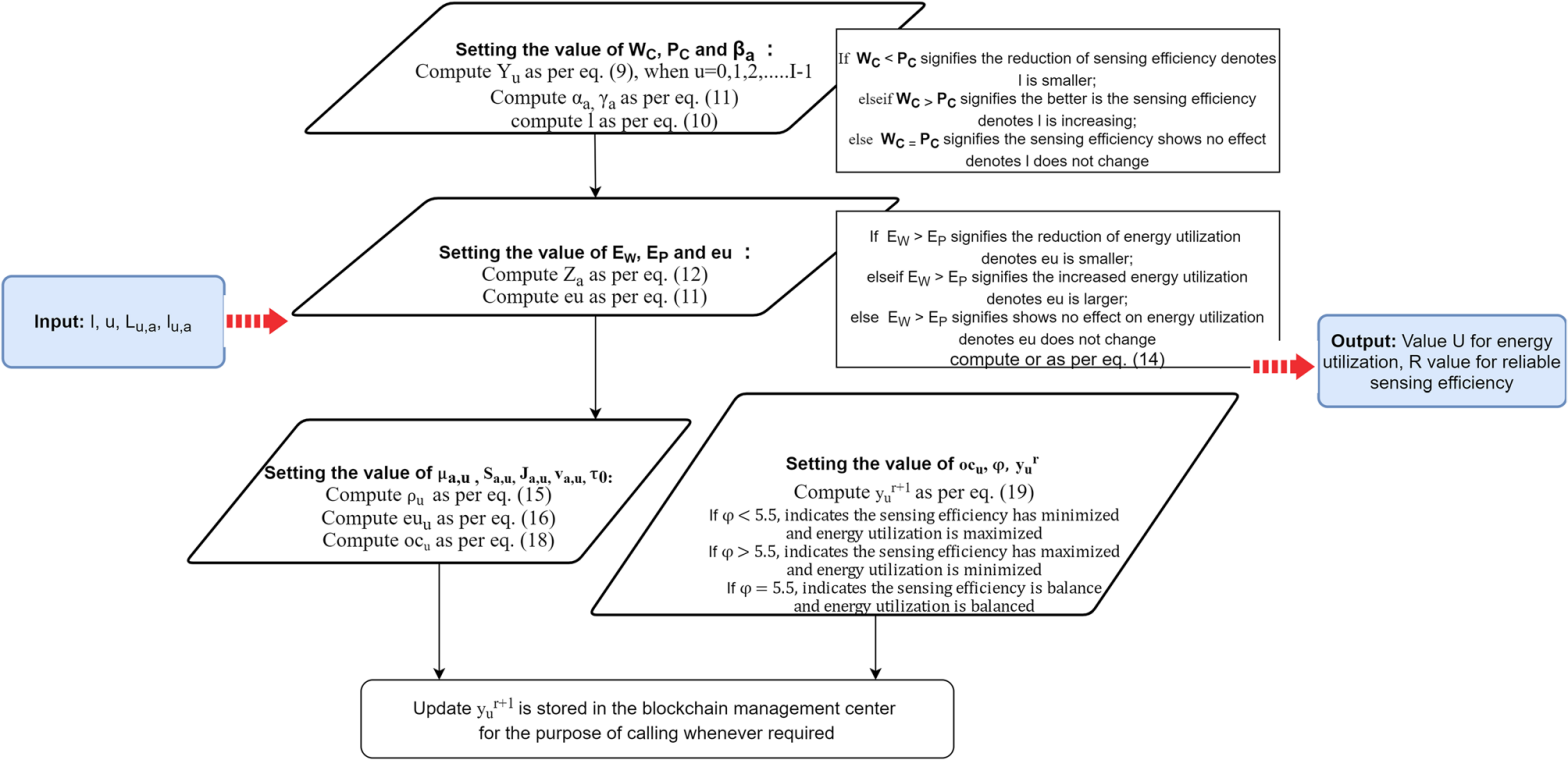
Flowchart for the evaluation and interpretation of nodes.

In the above Eq. (19), $y_u^r$ shows the nodes trust value in the *a*th sensing cycle for the *u*th node; $y_u^{r+1}$ shows the present nodes trust value for the $u_{th}$ node; $oc_u^r$ is the overall return value of the sensing cycle; the $\varphi$ denotes the value either 1 or 0. More the value of $\varphi$ gives better efficiency for energy utilization. The flowchart for the evaluation and interpretation of nodes is shown in Fig. 4.

The complexity of the proposed flowchart is O(I!) where O is denoted Big O Notation, I is the total number of nodes in the trust value. In the design flowchart, the main difficulty of blockchain-enabled CWN among the IoT devices shows that this article consists of the blockchain system, CWN’s and IoT devices. The FC is where users interact with the blockchain system. The IoT device provides node data to the FC, which searches the blockchain system for node data. The node then transmits verified by the private key to the FC, which validates if the sensing node has a matching private key pair. If that’s the case, send the node’s request to the blockchain system, and the blockchain system’s confirmation to the sensing node. The data verified by the sensing node can verify the identity of those taking part in CSS and guarantee that their message has not been tampered with. The steps to follow for the designing of CWN are:

- Firstly, check the sensing nodes in the CWN. It transmits the information of nodes and then requests for the identification of encryption to the FC.
- Secondly, examine the verification request to the blockchain management center.
- Thirdly, the blockchain management center returns the verification information to the FC and then returns the encrypted data to the sensing nodes of CWN.

## Discussion

Let us assume that the CWN changes from quantity ten to sixty whose main objective is to select the best location of the sensor nodes and choose the sensing sensors regarding the limitations on the possibility of identifying Id and possibility of triggering Tr. Simulation outcome displays the $\alpha = 1.5$ and $\gamma = 2.5$ and acquired $\alpha = 0.8$ and $\gamma = 1.2$ in which all the nodes simulations outcome comes out to be over 2000 iterations. This experiment has been implemented in the solidity tool for the blockchain and the tests were This study involves a workspace is shown in Table 1 with two Intel Xeon Gold6 128 3.7GHz CPUs, 512 GB of memory, and a Panasonic S16GB SDHC Memory Card to run VMware ESXi 6.7.0 bare metal virtualized system. There are 8 auxiliary nodes and 30 sensor nodes in the modeled CWN. In this simulation environment, the total number of tampered users (TU) are described in two different cases: the first case, in case there are 5 TU’s then the SNR is − 16d B for 1 TU working under SNR; the second case, in case there are 10 TU’s then the SNR is taken as − 18d B for 3 TU’s working under SNR.

**Table 1:** Simulation environment.

QoS metrics	Range
Intel Xeon Gold6	128.37GHz
Memory	512GB
Panasonic	S16GB SDHC
VMware	ESXi 6.7.0
Auxilary nodes	8
Sensor nodes	30

**Figure 5:**
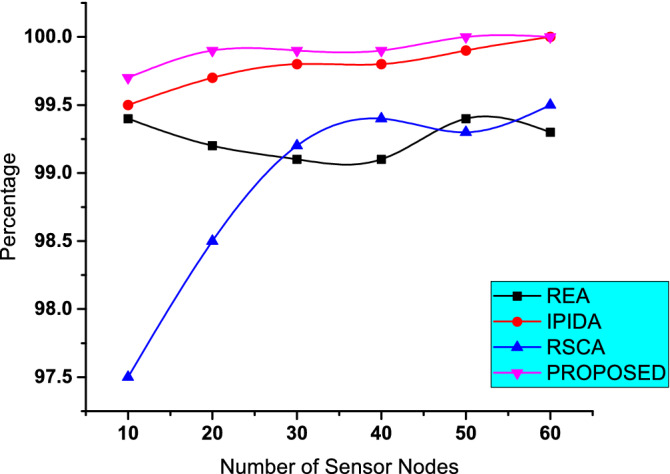
Percentage for finding the solution with $\alpha = 1.5$ and $\gamma = 2.5$ with varying number of nodes.

Authors have utilized the 5.2 MHz IEEE 802.15.4/ZigBee for the CSS communication which interacts with each other to sense the spectrum. For the performance evaluation, authors have assumed that the sensors that are located at the sensing field are 200m in length where the sensing nodes are uniformly dispersed and in the middle of CSS, FC is situated. In this article, while taking the evaluation and interpretation of nodes flowchart into account, the total number of TU’s are taken as 10. Therefore, to enhance the sensing efficiency of the nodes the comparison for various sensing has been discussed in this article.

The licensed users are based on the location which is placed on the edge of the circular area; the total number of nodes required is assumed as 30 (10 nodes having SNR = − 16 dB, − 18 dB and − 12 dB) respectively for all the nodes. The total TU’s are 3 when the SNR is − 18 dB. In the situation of four TU’s in CWN, the flowchart in this article’s identification Id is greater than the classical method under the same possibility of triggering Tr. That’s because the flowchart in this article takes into account the environment and other variables, and the CWN is more resilient and anti-attack competent. The node information may be monitored in real-time in any location, and blockchain technology encrypts the node information. The FC can request that high-performing nodes engage in CSS, ensuring that sensing performance is always improved.

In this article, various algorithms have been compared with the proposed work. In the Reduced energy Algorithm (REA), Sensors are ordered in increasing order as per their distances from the FC in this approach, and nodes with the shortest distances are chosen to sense the channel such that Id $> \gamma$ is fulfilled. There are fewer sensing nodes than I. If REA can identify nodes that meet the Id $> \gamma$ criterion, then its solution is close to optimum since it selects nodes with the shortest distances from the FC. In the Increased possibility of Identification Algorithm (IPIDA), Sensors are ordered in decreasing order as per their $Id_u$ in this flowchart. As a result, the nodes with the highest $Id_u$ are chosen, and the selection nodes are less than I, satisfying the $Id_u > \gamma$ condition. If there is a response for the algorithm, IIPDA discovers it. The randomized sensor choosing algorithm (RSCA) selects sensors for CSS at random, with the number of choosing nodes being less than I. This algorithm is the simplest to use to identify a solution for the problems.

**Figure 6:**
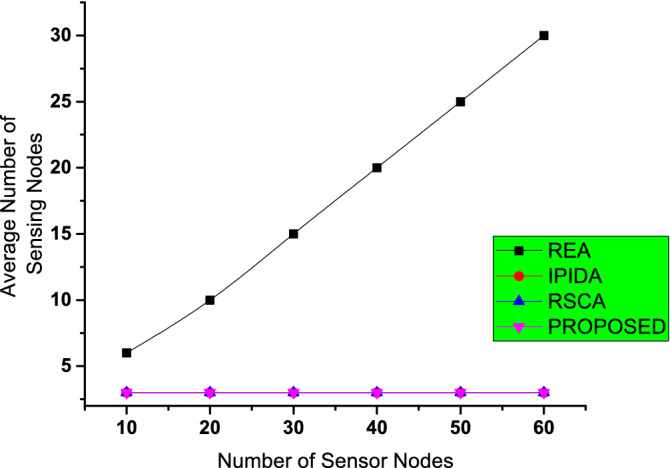
Average number of sensing nodes with $\alpha = 1.5$ and $\gamma = 2.5$ with varying number of nodes.

The REA algorithm when the number of nodes rises, the energy consumed in the network is minimized because the nodes comes closer to one another and their distance will be decreased while transferring the data as shown in Fig. 6. But on the other side IIDPA uses the less number of nodes. Our proposed algorithm and REA are closer to the IIDPA which displays that our proposed algorithm consumes less energy while maximising the number of nodes.

Figure 5 shows the success rate of finding a solution for several methods that meet $Id > \gamma$. This statistic for each method demonstrates the algorithms’ capacity to locate the answer when the problem conditions are met. It is shown that the REA method is compatible with the IIPDA algorithm, implying that if the problem has a solution, our proposed scheme can locate it. On the other hand, probably, REA and RSCA will not discover a solution to the issue while it has one.

The average number of sensing nodes for various methods is displayed in Fig. 6. IIPDA employs the smallest number of nodes possible. IIPDA and our algorithm and REA are extremely similar. This shows that our approach uses the least amount of energy while keeping $Id > \gamma$ constant. The RSCA algorithm uses the most sensing nodes.

**Figure 7:**
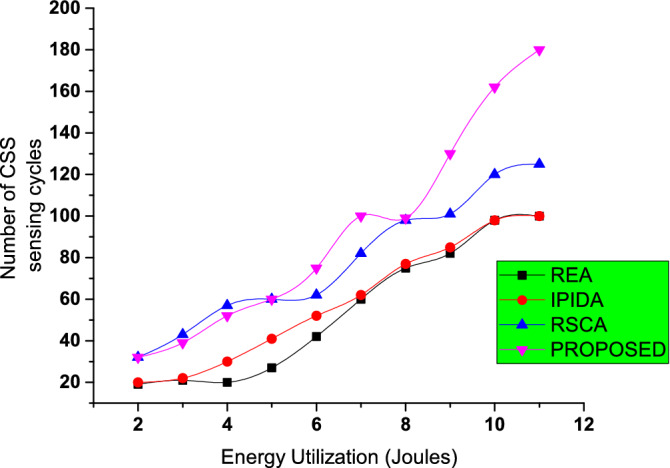
Comparison of energy utilization with existing algorithms.

Figure 7 shows that when the number of sensing cycles surpasses 60, the energy consumption of the method in this paper is much lower than the other three main techniques. The more sensing cycles there are, and the longer the sensing duration is, the more energy is saved. CWN will be greatly aided in extending the operating life. That’s because the methodology in this work can always choose the node with the best performance to participate in CSS in real-time, saving more energy than the conventional method when sensing efficiency is preferable.

## Conclusion

This paper examines the concept of CWN for smart cities in detail. CSS sensing collaboration is critical for limiting the effects of shadowing and fading, as well as accurate sensing. Nevertheless, as the number of users grows, we spend lots of time observing, analyzing, and transmitting data. Furthermore, expanding the amount of spectrum sensing users does not enhance the system’s detection accuracy exponentially. As a result, it is not required to use all users at all times for spectrum sensing. So, when nodes sense data in practical implementation situations of CWN, there are generally major mistakes that cause the sensing values to depart from the normal range, or some nodes intentionally relay the erroneous data to the FC. As a result, this study offers the Evaluation and interpretation of nodes flowchart to address the security challenge of TU’s attack in CWN for smart cities. In this article, the authors have made a comparison with various existing algorithms based on the percentage of finding the solution of sensing nodes, the average number of sensing nodes and energy utilization. It has been found that the proposed methodology achieves 10% sensing efficiency in finding the solution for identification and triggering possibilities with the value of $\alpha = 1.5$ and $\gamma = 2.5$ with the varying number of nodes.

## Data Availability

All data generated or analysed during this study are included in this published article.
